# The Current Research Landscape of the Application of Artificial Intelligence in Managing Cerebrovascular and Heart Diseases: A Bibliometric and Content Analysis

**DOI:** 10.3390/ijerph16152699

**Published:** 2019-07-29

**Authors:** Bach Xuan Tran, Carl A. Latkin, Giang Thu Vu, Huong Lan Thi Nguyen, Son Nghiem, Ming-Xuan Tan, Zhi-Kai Lim, Cyrus S.H. Ho, Roger C.M. Ho

**Affiliations:** 1 Institute for Preventive Medicine and Public Health, Hanoi Medical University, Hanoi 100000, Vietnam; 2 Bloomberg School of Public Health, Johns Hopkins University, Baltimore, MD 21205, USA; 3 Center of Excellence in Evidence-Based Medicine, Nguyen Tat Thanh University, Ho Chi Minh City 700000, Vietnam; 4 Institute for Global Health Innovations, Duy Tan University, Da Nang 550000, Vietnam; 5 Centre for Applied Health Economics, Griffith University, Queensland 4111, Australia; 6 Department of Psychological Medicine, Yong Loo Lin School of Medicine, National University of Singapore, Singapore 119074, Singapore; 7 Department of Psychological Medicine, National University Hospital, Singapore 119074, Singapore; 8 Center of Excellence in Behavioral Medicine, Nguyen Tat Thanh University, Ho Chi Minh City 700000, Vietnam; 9 Institute for Health Innovation and Technology (iHealthtech), Singapore 119074, Singapore

**Keywords:** artificial intelligence, cerebrovascular, heart diseases, bibliometrics, scientometrics

## Abstract

The applications of artificial intelligence (AI) in aiding clinical decision-making and management of stroke and heart diseases have become increasingly common in recent years, thanks in part to technological advancements and the heightened interest of the research and medical community. This study aims to provide a comprehensive picture of global trends and developments of AI applications relating to stroke and heart diseases, identifying research gaps and suggesting future directions for research and policy-making. A novel analysis approach that combined bibliometrics analysis with a more complex analysis of abstract content using exploratory factor analysis and Latent Dirichlet allocation, which uncovered emerging research domains and topics, was adopted. Data were extracted from the Web of Science database. Results showed topics with the most compelling growth to be AI for big data analysis, robotic prosthesis, robotics-assisted stroke rehabilitation, and minimally invasive surgery. The study also found an emerging landscape of research that was centered on population-specific and early detection of stroke and heart disease. Application of AI in health behavior tracking and improvement as well as the use of robotics in medical diagnostics and prognostication have also been found to attract significant research attention. In light of these findings, it is suggested that the currently under-researched issues of data management, AI model reliability, as well as validation of its clinical utility, need to be further explored in future research and policy decisions to maximize the benefits of AI applications in stroke and heart diseases.

## 1. Introduction

Cardiovascular disease, which includes heart diseases and stroke, [1] accounts for 366 million healthy life years lost across all age groups and genders. Individually, ischemic heart disease and stroke are two of the five leading causes of healthy years lost globally. [2] The physiological, social and psychological impact of these cardiovascular diseases vary across populations and individuals. Fortunately, there is an array of treatment options available, but timely diagnosis, appropriate interpretation of investigation results and apt patient selection for the various intervention methods are essential.

Artificial intelligence (AI) has been a disruptive innovation in the world of health and medicine. Not only has it been applied for medical research, but AI can also provide algorithmic solutions in clinical settings to aid in the diagnosis, prognosis, treatment and visual pattern recognition software in fields such as radiology to aid in the interpretation of imaging. Significant attention is now turning to the potential of AI in the medical field. According to a 2019 bibliometric study, the number of studies on AI applications in medicine has tripled in the past three years, with heart diseases and stroke as two of the top three topics of interest [3]. 

Various techniques such as robotics, machine learning, and natural language processing have been applied to the study of these cardiovascular diseases. Some cutting edge applications of machine learning models include: predicting the presence of a high-risk plaque or an absence of coronary atherosclerosis, using biomarkers in patients with suspected coronary artery disease [4], selecting suitable elderly patients for endovascular therapy to reduce intracerebral hemorrhage after thrombectomy [5], grading of coronary artery stenosis and extent of myocardial ischemia [6,7,8,9,10], as well as stroke lesion outcome prediction [11,12,13,14,15,16,17,18]. Some authors have explored the potential of image-based AI applications in the scoring of non-contrast computerized tomography scans [19,20] as well as machine learning in the prediction of mortality in coronary artery disease and heart failure patients based on echocardiography [21]. The potential for AI to aid in clinical decision-making and management of stroke and heart diseases is manifold and ever expanding.

As this area of interest grows, it is important to understand the current research landscape and trajectory. This study aims to appraise extant literature through bibliographic analysis to uncover global trends and developments in the use of AI for stroke and heart disease.

## 2. Materials and Methods 

### 2.1. Search Strategy

We searched and retrieved all papers published in the period from 1991 to 2018 related to artificial intelligence in stroke and heart diseases on the Web of Science, which is an online database covering the largest proportion of the peer-reviewed literature in this field. The full search strategy has been presented elsewhere [3]. In this analysis, we selected all documents of the retrieved data on AIs that related to stroke and heart diseases.

### 2.2. Data Extraction

**Figure 1:**
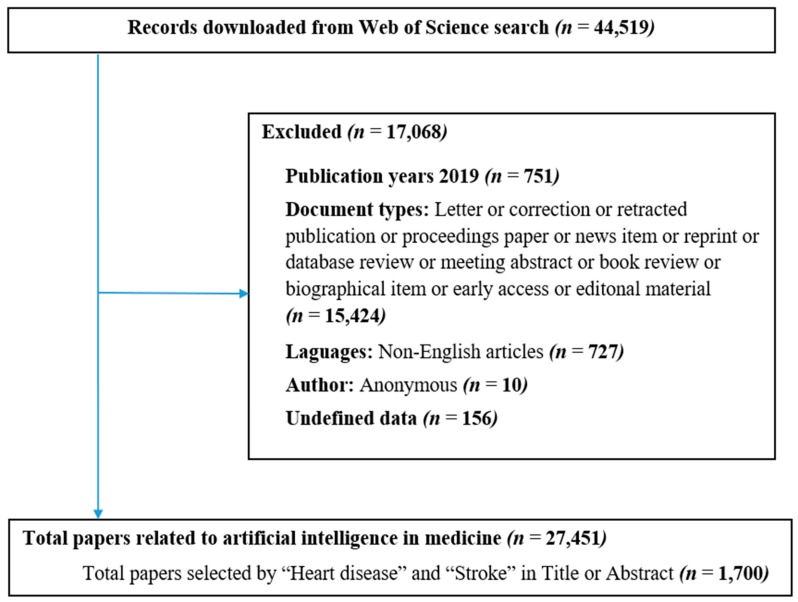
Paper selection process.

We downloaded all data from the Web of Science (WoS) database in .txt format, including all paper information such as authors’ names, paper title, journal name, keywords, institutional affiliations, frequency of citation, subject category, and abstracts. All of these data were entered into a Microsoft Excel file to check data error. A process of standardization was carried out by two researchers to bring together the different names of an author. Subsequently, all downloaded data was filtered by excluding papers which were: (1) not original articles and reviews, (2) not about stroke and heart diseases and AIs, and (3) not in English. Any conflict was solved by discussion (Figure 1). The combined dataset was transferred into Stata for further analysis.

### 2.3. Data Analysis

Data were analyzed based on basic characteristics of publication (number of authors, publication years, main category), keywords (most common keywords and co-occurrence keywords), citations, usages (the number of times a paper is downloaded), and abstracts. After downloading and extracting the data, we applied descriptive statistical analysis to calculate total citations by country and intercountry collaboration. A network graph illustrated the connection among countries based on co-authorship, along with an author keyword co-occurrence network and country network. VOSviewer (version 1.6.8, Center for Science and Technology, Leiden University, the Netherlands) was used to establish a co-occurrence network and a country network. For content analysis of the abstracts, we applied exploratory factor analysis with loading of 0.4 to identify research domains emerging from all content of the abstracts. Haberman distance was utilized to identify the research topics that most frequently co-occurred or were related to each other. Latent Dirichlet allocation (LDA) was used to classify papers into corresponding topics [22,23,24,25,26]. The summary of analytical techniques for each data type is presented in Table 1.

## 3. Results

There has been a rapid increase in the number of studies regarding the application of AI in stroke and heart disease research during 1991–2018. In particular, the total number of papers published in the last five years accounted for over 65% of the total papers for the whole period. More recently published papers also have significantly higher total usage (the number of times a paper is downloaded) both within the last six months and the last five years (Table 2). 

In Table 3, we examined the study settings mentioned in the abstracts of publications. The highest proportion of the studies were conducted in the United States (44.1%), much higher than that of the second most popular country (Ireland at 10.2%). The top ten countries by study setting, which accounted for over 80% of the total studies with available setting information, saw the domination of developed nations, except for India, which, on the other hand, is known for research strength in information systems and healthcare.

**Figure 2:**
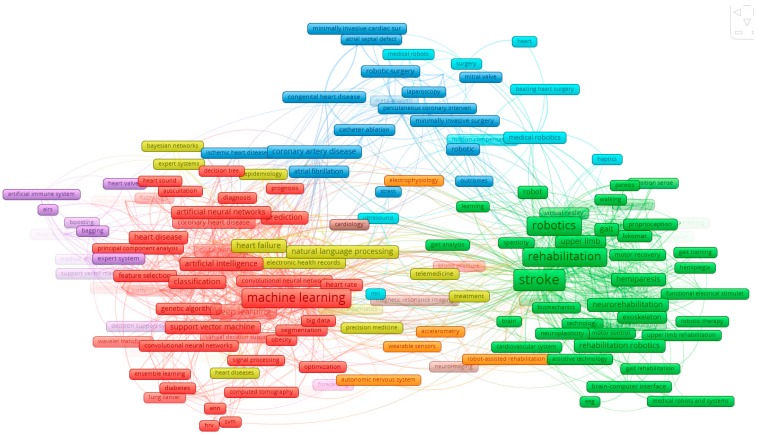
Co-occurrence of the most frequent author’s keywords. Note: the colors of the nodes indicate principle components of the data structure; node size was scaled to keyword occurrences; the thickness of the lines was drawn based on the strength of the association between two keywords. (ANN: artificial neural network; EEG: electroencephalogram; HRV: heart rate variability; MRI: magnetic resonance imaging; SVM: support vector machine).

We analyzed paper keywords and abstracts and presented the network of keyword co-occurrence of 200 of the most frequent keywords that appeared together at least five times (Figure 2). Several major clusters can be seen from this network, showing how words that co-occur often appear under a common broader topic. In particular, Cluster 1 (red) contains words relating to most common machine learning techniques and models being applied in heart disease management; Cluster 2 (green) covers the use of robotics in stroke rehabilitation; Cluster 3 (blue) refers to the application of AI in surgical intervention for heart problems; and Cluster 4 (yellow) represents AI application in medicine and care for heart disease.

**Figure 3:**
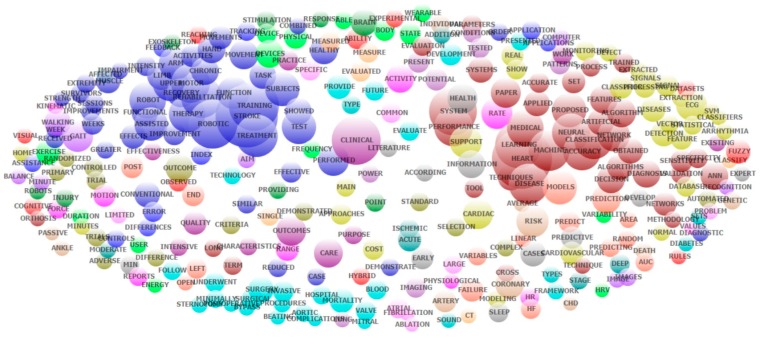
Co-occurrence of the most frequent topics that emerged from the exploratory factor analysis of abstracts contents. (ANN: Artificial Neural Network; AUC: area under the curve; CHD: coronary heart disease; CT: computed tomography; ECG: electrocardiogram; HF: heart failure; HR: heart rate; HRV: heart rate variability; SVM: support vector machine).

Table 4 presents the results of the exploratory factor analysis of all abstracts’ contents. The most common research domains regarding AI applications in stroke and heart diseases in 1991–2018 have been rehabilitation and prediction of therapy outcome for stroke patients (for example, domain numbers 1, 5, 8, 11 in Table 3); machine learning techniques and models (for example, domain numbers 2 and 6); surgical intervention for heart diseases (for example, domain numbers 3 and 12). The application of AI in health behavior tracking/improvement has also been an emerging research domain within stroke and heart diseases (for example, domain number 29). Figure 3 provides a visualization of the top research domains listed in Table 4.

In Table 5, we present the research topics that were constructed using LDA. The labels of the topics were manually annotated by scrutinizing the most frequent words and titles for each topic. Topics with the highest volumes of publications included: (1) general reviews of AI-related techniques and models for application in health studies (Topics 1 and 2 in Table 4); (2) AI application in cardiac surgery (Topics 3 and 6); (3) robotics application in stroke rehabilitation (Topics 4 and 5); (4) AI assistance in diagnosis/screening and other population-specific investigations (Topics 7–10). Interestingly, LDA analysis of all paper contents has revealed an emerging research landscape of research that centered on population-specific and early detection of stroke and heart diseases (enabled by AI advancements) that otherwise would be overlooked by keyword and abstract analysis. 

**Figure 4:**
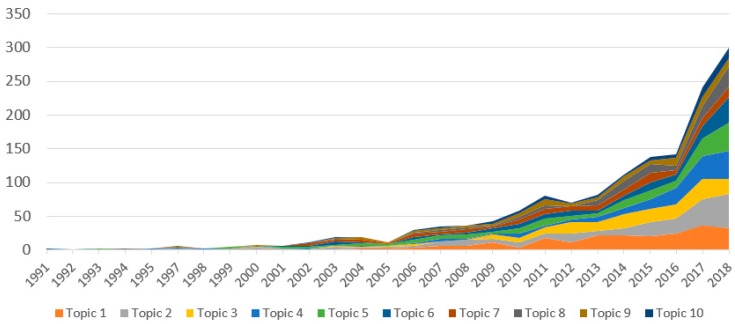
Changes in applications of AI to stroke and heart disease research during 1991–2018.

The changes in research productivity over time is illustrated in Figure 4. It shows a significant increase in the number of studies of all the most popular topics in the last five years, especially since 2016. The topics with the most compelling growth have been Topic 2 (AI for big data analysis), Topic 4 (robotic prosthesis), Topic 5 (robotics-assisted stroke rehabilitation) and Topic 6 (minimally invasive surgery).

**Figure 5:**
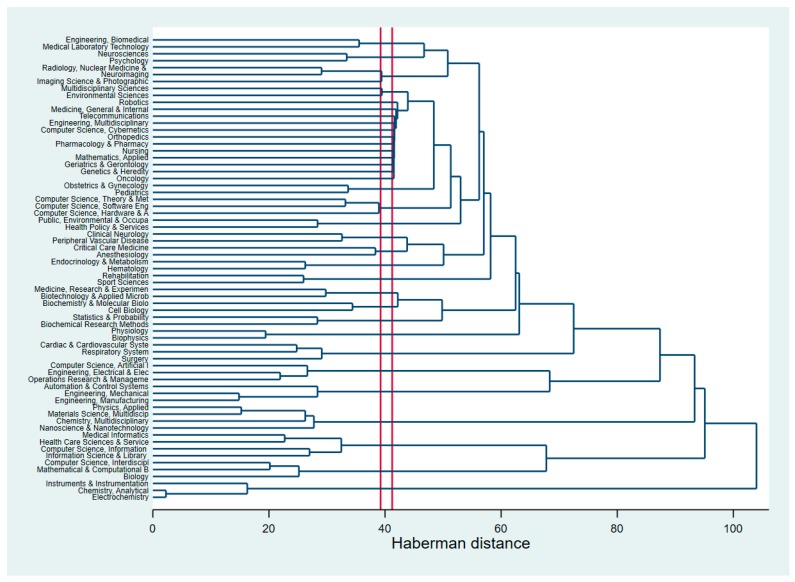
Dendrogram of research areas using the WoS classifications.

We also attempted to analyze research clustering by the research areas classified by WoS. Figure 5 (dendrogram) shows how closely linked these areas are with regard to AI application in stroke and heart diseases. The horizontal axis of the dendrogram represents the distance (Haberman distance) or dissimilarity between research disciplines. The vertical axis represents the research disciplines based on WoS classification. The smaller the distance, the closer the disciplines cluster together and the higher their similarity. The most striking feature is possibly the connection between robotics and a range of aspects including medicine, care and other medical fields (for instance, oncology, geriatric, genetics, etc.). The clustering of other research areas is similar to that found in the analysis of authors’ keywords, abstracts, and content; for example, cardiac surgery with AI/computer science. 

Another visualization of the clustering of research disciplines (based on WoS classification) can be found in Figure S1. The main clusters include: (1) AI-enabled tools and models applied in heart surgery; (2) AI-assisted applications (including neuroscience/neuroimaging) in stroke rehabilitation, (3) multidisciplinary research (including biology/chemistry/ biophysics).

## 4. Discussion

The results of our study indicate a growing interest regarding the application of AI in the management of stroke and heart disease. Such research has gained greater traction in recent years, as evidenced by significantly higher indices of article publication and usage in the last five years. Whilst there is a rapid increase in publications pertaining to AI in the management of stroke and heart disease, this study, to the best of our knowledge, can be considered the first in providing a macroscopic organizational framework of existing literature on the subject matter. The insight gained from this endeavor will hopefully influence future developments and the direction of this field.

Advances in technology, infrastructure and knowledge have allowed information technology and engineering to progress by leaps and bounds. Highly sophisticated, technologically advanced and computationally demanding solutions are becoming increasingly practical and have allowed an era of novel and innovative solutions. This development is exceptionally conducive for the growth of fields like AI and likely accounts for the unprecedented expansion of scientific literature on AI in managing stroke and heart disease (Table 2).

To date, the foci of progress has been centered on developed countries, most notably the US, which contributes 44.1% of publications. This is not unexpected as the requirements for AI to flourish meaningfully are stringent and developing countries may need more time to develop such capabilities. Over time, it will be interesting to see how the landscape evolves with greater involvement of countries like India and China who contribute 5.7% and 0.8% of publications, respectively, despite being the most populous countries in the world. 

Our keyword analysis (Figure 2), exploratory factor analysis (Table 4) and LDA (Table 5) are corroborative and identify machine learning and modeling, stroke rehabilitation, and cardiac surgery as the most dominant research domains. Within each of these domains are topics of particular interest. In machine learning and modeling, neural networks and support vector machines for medical diagnosis, prognosis, and classification are most commonly mentioned and account for 26.1% of publications. Machine learning allows virtual machines to learn from data, establishing relationships and improving their capabilities autonomously without explicit programming [27]. With massive medical databases, parameters, and outcomes, machine learning is perfectly suited for the task of sieving through data to detect patterns that aid in the diagnosis of conditions like angina from clinical notes [28], predicting mortality of intracerebral hemorrhage [29] or identifying heart failure patients from electronic medical records [30]. In stroke rehabilitation, robotics for prosthesis or training in rehabilitation, as well as prediction of recovery, is most frequently studied and accounts for 22.8% of publications. In cardiac surgery, the main interest is in minimally invasive robotic surgery for valve repair or coronary artery disease and accounts for 20.4% of publications. Principal component analysis displays a strong relationship between AI, heart surgery and stroke, demonstrating the current development of the field. 

Several trends were noted in the current study. Topics seeing the most compelling growth are that of AI for big data analysis, robotic prosthesis, robotic-assisted stroke rehabilitation, and minimally invasive surgery. The application of AI in health behavior tracking and improvement is also starting to emerge in the management of stroke and heart disease. These trends are positive indicators that the current hardware and software are becoming more able to support cutting edge projects that were previously limited by technology [31]. The LDA of all papers’ content also suggests that there is an emerging landscape of research that is centered on population-specific and early detection of stroke and heart disease.

The rise of AI robotics and AI models in stroke and heart disease management has far-reaching clinical implications that may be realized in the near future. As the current cutting-edge robotic technology translates to the healthcare market, clinicians and patients alike will see an increase in sophisticated surgical and rehabilitative technology. To patients, better neuro-prosthesis will allow better function and quality of life improvements after cerebrovascular insult. To physiotherapists, greater capabilities of robotic devices will mean better, faster, safer and more convenient rehabilitation. For clinicians, the increase in the prevalence and capabilities of smart wearable devices may greatly impact management guidelines of chronic cerebrovascular and heart diseases. To cardiac surgeons, new robotic tools and surgical techniques will enable more minimally invasive approaches to surgical heart disease. These extrapolations are modest, and it is reasonable to imagine the effects of AI robotics as even more profound. AI models will have an equal if not greater impact on the future of stroke and heart disease management. With greater refinement, these models may be powered to enable rapid screening, diagnosis, and prognostication of stroke and cardiovascular disease. Its application will allow early detection of disease, identification of high-risk populations and initiation of treatment. Prognosticative tools will advise on the extent of recovery, allowing clinicians to set better rehabilitation targets and manage expectations of patients and their families.

AI in healthcare faces a distinct set of challenges that transcend medical specialties. Themes of particular relevance include data management, clinical utility, and reliability of models [32,33,34]. On the topic of data management, the use of health data to develop and validate models is a delicate issue. AI models will require access to large databases of health records in order to function optimally. This inadvertently exposes data management systems to a very real threat of compromised confidential data. Developments to address this concern are not evident in the bibliometric analysis and could represent an area which needs greater attention. The clinical utility and reliability of models is another issue which can be addressed further. To start, machine learning in AI models has an inherent trade-off between the complexity of models and generalizability to new data sets [34,35]. Cerebrovascular and cardiovascular disease also faces the challenge of finding large unbiased sources of phenotypic data for disease characterization [35]. This problem of model reliability is most commonly addressed through validation with independent datasets (Table 4; items 35 and 38) and it has enjoyed some success in small populations [36,37,38]. However, it is noted that current state-of-the-art methods are still not robust or accurate enough for large scale clinical application [12]. Improving data quality and expanding data set sizes may alleviate these problems but perhaps a more useful direction would be to better translate the clinical utility of these models in select populations. It is notable that, from current literature and our bibliographic analysis, the clinical utility of AI approaches lack assessment and validation through large-scale, prospective cohort studies [10]. Studies in this area are not difficult to conduct and may offer immense practical value to clinicians. 

While great effort has gone into conducting this bibliometric analysis through an intensive summary of keywords and research patterns, there are some limitations of this study. This study only included English papers and may underreport trends and studies of research conducted in other languages. In addition, the publication type was restricted to peer-reviewed publications, and this may influence the thoroughness of the analyzed results.

## 5. Conclusions

In conclusion, the findings of our study depict a recent sharp rise of research production on the topic of AI application in the management of stroke and heart disease. The prevailing research themes uncovered by our analysis demonstrated the growing utility of robotics in stroke rehabilitation, robotics in cardiac surgery and AI models for medical diagnostics and prognostication. These developments are clinically significant and will influence the future of stroke and heart disease management for multiple stakeholders. On the other hand, the study found that issues of data management, AI model reliability and validation of clinical utility of AI models have yet to be discussed extensively in the existing literature. Thus, for AI applications to realize their full capabilities, the study suggested that future research and policy decision-making processes should consider further exploring and resolving these issues.

## Figures and Tables

**Table 1:** Analytical techniques and outcomes of each data type.

Type of Data	Unit of Analysis	Analytical Methods	Presentations of Results
Keywords, Countries	Words	Frequency of co-occurrence	Map of keywords clusters
Abstracts	Words	Exploratory factors analyses	Top 50 constructed research domains Clustering map of the landscapes constructed by these domains.
Abstracts	Papers	Latent Dirichlet allocation	10 classifications of research topics
WoS classification of research areas	WoS research areas	Haberman distance	Dendrogram of research disciplines

**Table 2:** General characteristics of publications.

Year Published	Total Number of Papers	Total Citations	Mean Cite Rate per Year	Total Usage in the Last 6 Months	Total Usage in the Last 5 Years	Mean Use Rate in the Last 6 Months	Mean Use Rate in the Last 5 Years
2018	358	345	0.96	1968	3289	5.50	1.84
2017	273	1571	2.88	903	4139	3.31	3.03
2016	157	1149	2.44	287	2542	1.83	3.24
2015	152	1720	2.83	196	2424	1.29	3.19
2014	131	1884	2.88	136	2193	1.04	3.35
2013	100	1725	2.88	75	1819	0.75	3.64
2012	80	1903	3.40	85	1480	1.06	3.70
2011	91	3086	4.24	128	1724	1.41	3.79
2010	64	1827	3.17	42	708	0.66	2.21
2009	54	2481	4.59	58	859	1.07	3.18
2008	44	1751	3.62	32	483	0.73	2.20
2007	39	2032	4.34	25	438	0.64	2.25
2006	39	1956	3.86	33	503	0.85	2.58
2005	15	694	3.30	10	136	0.67	1.81
2004	23	650	1.88	6	75	0.26	0.65
2003	21	1373	4.09	20	222	0.95	2.11
2002	13	243	1.10	5	27	0.38	0.42
2001	8	304	2.11	3	39	0.38	0.98
2000	8	672	4.42	9	89	1.13	2.23
1999	8	494	3.09	4	65	0.50	1.63
1998	3	26	0.41	1	1	0.33	0.07
1997	8	926	5.26	13	122	1.63	3.05
1995	3	25	0.35	0	3	0.00	0.20
1994	2	51	1.02	0	2	0.00	0.20
1993	3	51	0.65	0	2	0.00	0.13
1992	1	10	0.37	0	1	0.00	0.20
1991	2	4	0.07	0	2	0.00	0.20

**Table 3:** Number of papers by countries as study settings.

No.	Country Settings	Frequency	%	No.	Country	Frequency	%
1	United States	108	44.1%	19	Czech	2	0.8%
2	Ireland	25	10.2%	20	France	2	0.8%
3	Italy	15	6.1%	21	Netherlands	2	0.8%
4	India	14	5.7%	22	Singapore	2	0.8%
5	Australia	9	3.7%	23	United Arab Emirates	2	0.8%
6	Japan	8	3.3%	24	Antarctica	1	0.4%
7	Taiwan	6	2.4%	25	Brazil	1	0.4%
8	Spain	5	2.0%	26	Bulgaria	1	0.4%
9	United Kingdom	5	2.0%	27	Egypt	1	0.4%
10	Germany	4	1.6%	28	Greece	1	0.4%
11	Israel	4	1.6%	29	Jordan	1	0.4%
12	Switzerland	4	1.6%	30	Malaysia	1	0.4%
13	Iran	3	1.2%	31	Mexico	1	0.4%
14	Poland	3	1.2%	32	New Zealand	1	0.4%
15	Saudi Arabia	3	1.2%	33	Pakistan	1	0.4%
16	Austria	2	0.8%	34	Sweden	1	0.4%
17	Canada	2	0.8%	35	Tunisia	1	0.4%
18	China	2	0.8%	36	Turkey	1	0.4%

**Table 4:** Top 50 research domains that emerged from the exploratory factor analysis of all abstract content.

No.	Name	Keywords	Eigen-Value	Freq.	% of Cases
1	Fugl-Meyer; upper	Fugl; meyer; upper; motor; rehabilitation; Fugl-Meyer (FMA); limb; extremity; impairment; arm; reaching; improvements; weeks; therapy; stroke	19.3	758	53.8%
2	Support vector; machine (SVM)	Vector; svm; support; feature; classification; machine; heart rate variability (HRV)	6.8	385	48.8%
3	Coronary artery bypass; surgery	Bypass; surgery; postoperative; endoscopic; surgical; invasive; procedures; left; underwent; coronary; times	4.8	273	33.3%
4	Blood pressure (BP)	Pressure; blood; bp; tilt	3.8	63	10.8%
5	Flexion; joint	Flexion; joint; elbow; passive; motion; movements; healthy; range	3.4	244	34.1%
6	Neural network	Neural; artificial; network; artificial neural network (ANN); networks	3.3	256	27.8%
7	Predict	Area under the curve (AUC); Rheumatoid factor (RF); random; predicting; predictive; predict	3.2	134	22.6%
8	Gait; walking	Gait; walking; lokomat; practice; phase; training	3.0	192	33.1%
9	Machine learning; heart disease	Machine; learning; disease; accuracy; classification; prediction; risk; heart	2.9	811	72.7%
10	Fuzzy; systems	Fuzzy; systems; expert; decision; problem; medical	2.8	224	39.1%
11	Sensitivity	Sensitivity; specificity; detection; predictive	2.6	137	21.3%
12	Mitral valve; repair	Valve; mitral; repair; underwent	2.5	62	10.0%
13	Brain; hand	Brain; hand; stimulation; plasticity; movements; functional; brain-computer interfaces (BCI)	2.5	197	29.4%
14	Randomized controlled; assisted	Controlled; randomized; assisted; conventional; improvement; functional; efficacy; treatment	2.4	386	49.9%
15	Assistance; finger	Assistance; finger; virtual; demonstrated; activities	2.4	106	19.7%
16	Image	Images; image; computed tomography (CT); deep	2.4	58	10.2%
17	Observed; effects	Observed; effects; week; post	2.3	129	23.4%
18	Sensor; healthcare	Sensor; healthcare; monitoring; framework	2.1	73	15.5%
19	Complications; respiratory	Complications; respiratory; cardiac	2.1	88	17.6%
20	Exercise; subjects	Exercise; subjects; peak; tilt	2.1	106	22.3%
21	State; applied	State; applied; field	2.1	104	21.5%
22	Atrial	Atrial; atrial fibrillation (AF); catheter; procedure	2.0	61	11.3%
23	Paper	Paper; presents; proposed; experimental	2.0	243	39.4%
24	Space; terms	Space; terms; values	2.0	62	13.4%
25	Coronary artery; carotid	Artery; coronary; carotid; myocardial; disease; risk	2.0	261	39.9%
26	Clinical	Clinical; recent	2.0	160	37.5%
27	Conditions; future	Conditions; future; tested; healthy	1.9	155	29.1%
28	Variables; models	Variables; models; selected; develop; predict	1.9	167	30.5%
29	Physical activity; wearable	Physical; wearable; devices; activity; technology	1.9	185	32.0%
30	Chronic	Chronic; combined; weeks; week	1.9	133	23.4%
31	Able; user	Able; user; process; tested; wearable	1.8	135	25.2%
32	Diabetes; classifier	Diabetes; classifier; ensemble; dataset; classifiers; cancer; problems	1.8	147	23.1%
33	Muscle; guidance	Muscle; guidance	1.8	29	6.8%
34	Parameters	Parameters; error	1.8	72	16.8%
35	Validation	Validation; cancer; lung	1.8	63	13.1%
36	Severe; visual	Severe; visual; feedback	1.8	66	13.7%
37	Mortality; failure	Mortality; failure; outcomes; myocardial; hospital	1.7	179	29.9%
38	Trained; set	Trained; set; sets; validation	1.7	125	23.1%
39	Propose; terms	Propose; terms; show	1.7	101	21.5%
40	End; task	End; task; position; measured	1.7	124	24.2%
41	Robot	Robots; robot; therapy; field; intensity	1.7	213	34.1%
42	Multiple; index	Multiple; index; sleep; events	1.7	92	19.4%
43	Patterns	Patterns; pattern; potential; duration	1.6	119	25.5%
44	Technique; diagnosis	Technique; diagnosis; techniques	1.6	128	26.8%
45	Stroke	Stroke	1.6	168	44.1%
46	Pre; post	Pre; post; effective	1.6	117	22.6%
47	Quality	Quality; life	1.6	70	15.0%
48	Provided; differences	Provided; differences; acute	1.6	86	18.6%
49	Development; role	Development; role; plasticity	1.6	76	16.3%
50	Electrocardiogram (ECG); signals; arrhythmia	Electrocardiogram (ECG); arrhythmia; database; frequency; signals; normal; classifiers; cardiac	1.6	225	34.9%

**Table 5:** Top 10 research topics classified by Latent Dirichlet allocation (LDA). AI = artificial intelligence.

Year	Research Areas	Frequency	Percent
Topic 1	Reviews of AI and robotics in healthcare	234	15.9%
Topic 2	AI for big data analysis (genetics, metabolic studies)	217	14.8%
Topic 3	Robotically-assisted cardiac surgery	170	11.6%
Topic 4	Robotic prosthesis	167	11.4%
Topic 5	Robotics-assisted stroke rehabilitation	167	11.4%
Topic 6	Minimally invasive surgery	130	8.8%
Topic 7	AI for medical diagnostics	118	8.0%
Topic 8	AI for population identification	110	7.5%
Topic 9	AI-assisted biometric assessment	90	6.1%
Topic 10	AI interpretation of medical investigations	66	4.5%

## Supplementary Materials

The following are available online at https://www.mdpi.com/1660-4601/16/15/2699/s1, Figure S1: The coincidence of research areas using the WoS classifications (principle component analysis).
