# Supplementary material for: The Current Research Landscape of the Application of Artificial Intelligence in Managing Cerebrovascular and Heart Diseases: A Bibliometric and Content Analysis
Source: Int J Environ Res Public Health. 2019 Jul 29;16(15):2699. doi: 10.3390/ijerph16152699 (PMC6696240; doi:10.3390/ijerph16152699)
Supplement: Supplementary file 1 [file ijerph-16-02699-s001.pdf]

APPENDIX

Figure S1. The coincidence of research areas using the WoS classifications (Principle component analysis).

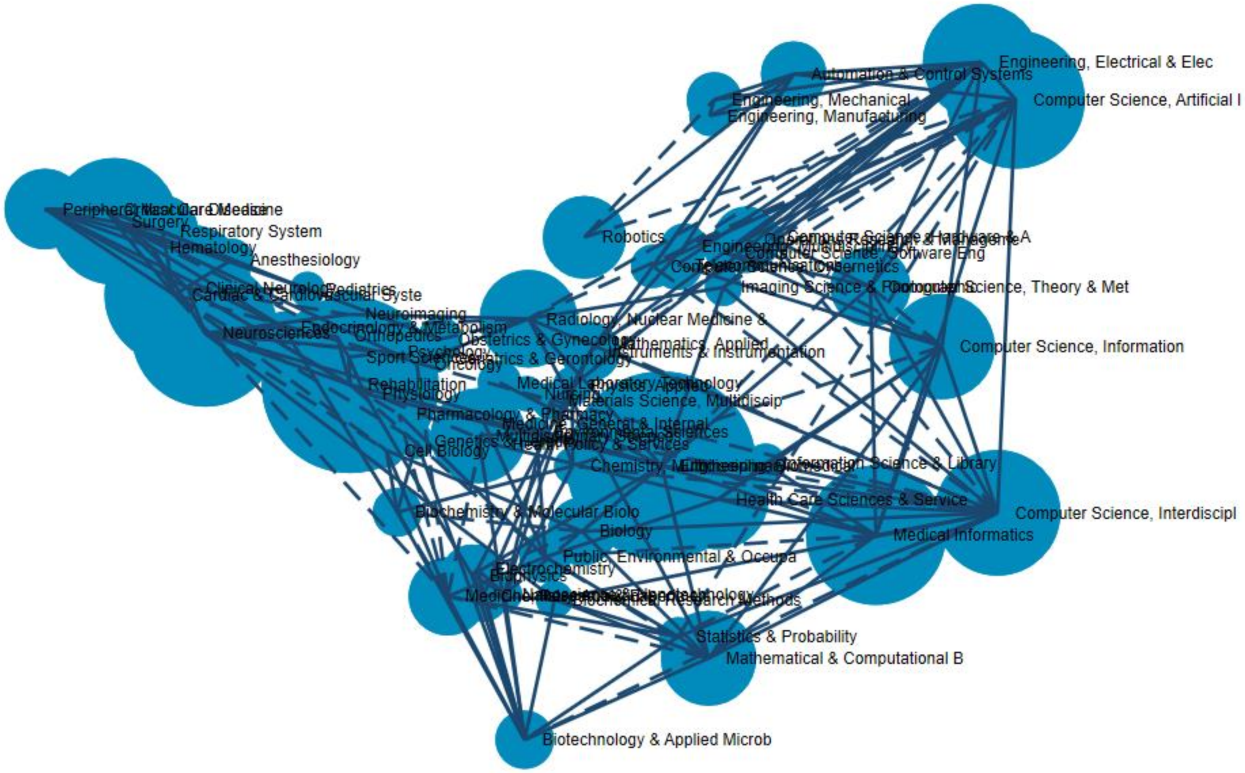

Each node shows the research area and its size represents the number of papers related to this discipline. A straight line means that the relationship is strong; while a dashed line shows the weak relationship.
